# AES/GRG5: More Than Just a Dominant-Negative TLE/GRG Family Member

**DOI:** 10.1002/dvdy.22439

**Published:** 2010-11

**Authors:** Brandon Beagle, Gail VW Johnson

**Affiliations:** 1 Deparkments of Anesthesiology and Pharmacology and Physiology, University of RochesterRochester, New York; 2 Department of Cell Biology, University of Alabama at BirminghamBirmingham, Alabama

**Keywords:** AES, GRG5, TLE/GRG, transcription factor, HDAC

## Abstract

The human Transducin-like Enhancer of Split (TLE) and mouse homologue, Groucho gene-related protein (GRG), represent a family of conserved non-DNA binding transcriptional modulatory proteins divided into two subgroups based upon size. The long TLE/GRGs consist of four pentadomain proteins that are dedicated co-repressors for multiple transcription factors (TF). The second TLE/GRG subgroup is composed of the Amino-terminal Enhancer of Split (AES) in humans and its mouse homolog GRG5 (AES/GRG5). In contrast to the dedicated co-repressor function of long TLE/GRGs, AES/GRG5 can both positively or negatively modulate various TF as well as non-TF proteins in a long TLE/GRG-dependent or -independent manner. Therefore, AES/GRG5 is a functionally dynamic protein that is not exclusively defined by its role as a long TLE/GRG antagonist. AES/GRG5 may function in various developmental and pathological processes but the functional characteristics of endogenous AES/GRG5 in a physiologically relevant context remains to be determined. *Developmental Dynamics 239:2795–2805, 2010*. © 2010 Wiley-Liss, Inc.

## INTRODUCTION

The Groucho/TLE/GRG protein(s) are a family of non-DNA binding co-factors that can interact with and mediate the transcriptional activity of DNA-binding transcription factors (Fisher and Caudy, 1998; Chen and Courey, 2000; Gasperowicz and Otto, 2005; Cinnamon and Paroush, 2008; Jennings and Ish-Horowicz, 2008). This transcriptional regulatory family plays a critical role in numerous developmental processes including osteogenesis and neurogenesis (Muhr et al., 2001; Wang et al., 2002, 2004; Gasperowicz and Otto, 2005; Buscarlet and Stifani, 2007). The Groucho/TLE/GRG family was first identified in *Drosophilia* for which there exists a single Groucho protein (Chen and Courey, 2000; Gasperowicz and Otto, 2005). The human and mouse Groucho homologs termed Transducin-like Enhancer of Split (TLE) and Groucho gene-related protein (GRG), respectively consist of a family of proteins that can be divided into two distinct subgroups based upon their size (Chen and Courey, 2000; Bajoghli, 2007).

**Fig. 1:**
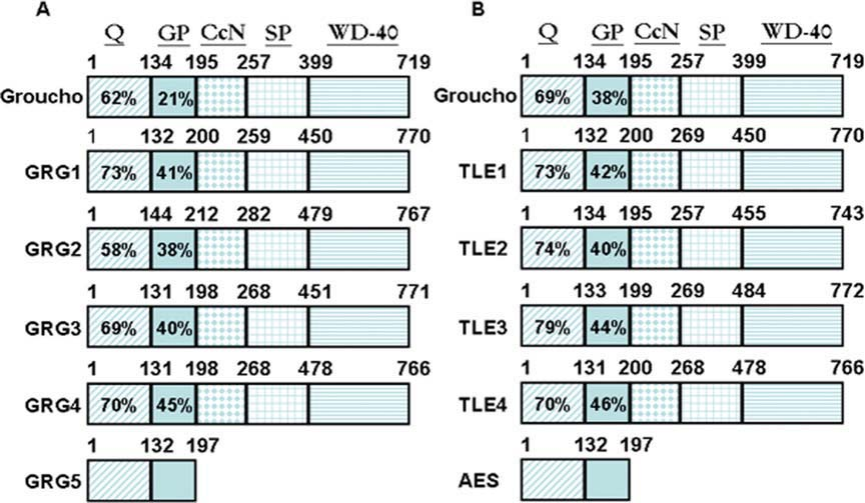
Structure and homology for the *Drosophilia* Groucho, TLE, and/or GRG protein family. The numbers above each box represent amino acid length for the domain identified at the top of the figure. **A,B:** The boxed numbers represent % homology for the Q or GP domain of *Drosophilia* Groucho or long GRG1-4 relative to GRG5 (A) *Drosophilia* Groucho or long TLE1-4 relative to AES(B). Percentage homology was calculated using global alignment with BLOSUM 62 scoring matrix. Amino acid sequence for the identified proteins are based upon the following NCBI accession numbers: *Drosophilia* Groucho (NP_733134), GRG1 (NP_035729.3), GRG2 (NP_062699.1), GRG3 (NP_033415.1), GRG4 (NP_035730.2), GRG5 (NP_034477.1), TLE1 (NP_005068.2), TLE2 (NP_003251.2), TLE3 (NP_005069.2), TLE4 (NP_008936.2) and AES (NP_001121.2).

The Long TLE/GRG subgroup consists of four pentadomain proteins (TLE1-4/GRG1-4) that function as dedicated co-repressors for multiple transcription factors (TF; Fig. 1; Fisher and Caudy, 1998; Chen and Courey, 2000; Gasperowicz and Otto, 2005; Bajoghli, 2007; Cinnamon and Paroush, 2008; Jennings and Ish-Horowicz, 2008; Jennings et al., 2008). The long TLE/GRG proteins are composed of a highly conserved amino-terminal Q domain (protein interaction and repression), followed by a GP domain (protein interaction and repression), CcN domain (nuclear localization signal, cdc2 and casein kinase II phosphorylation sites), an SP domain (protein interaction and repression) and a highly conserved WD40 domain (TF interaction; Miyasaka et al., 1993; Parkhurst, 1998; Chen and Courey, 2000; Brantjes et al., 2001; Courey and Jia, 2001; Gasperowicz and Otto, 2005; Bajoghli, 2007; Jennings and Ish-Horowicz, 2008). The Q domain mediates interaction with TFs such as Tcf/Lef-1 as well as the tetramerization of long TLE/GRG members, which is essential for their repressor function and interaction with TFs (Pinto and Lobe, 1996; Cavallo et al., 1998; Chen et al., 1998, 1999; Roose et al., 1998; Chen and Courey, 2000; Brantjes et al., 2001; Lopez-Rios et al., 2003; Song et al., 2004; Gasperowicz and Otto, 2005; Rave-Harel et al., 2005; Bajoghli, 2007; Orian et al., 2007; Sekiya and Zaret, 2007; Arce et al., 2009; Zhang et al., 2010). In addition to interacting with various TFs such as Runx2 (Thirunavukkarasu et al., 1998; McLarren et al., 2000; Wang et al., 2004), the GP domain of long TLE/GRGs interacts with histone deacetylases (HDAC), a mechanism by which long TLE/GRGs mediate transcriptional repression (Pinto and Lobe, 1996; Chen et al., 1999; Choi et al., 1999; Chen and Courey, 2000; Brantjes et al., 2001; Courey and Jia, 2001; Yochum and Ayer, 2001; Gregoretti et al., 2004; Daniels and Weis, 2005; Gasperowicz and Otto, 2005; Ye et al., 2009). The long TLE/GRG proteins can also mediate repression by blocking interaction between co-activators and TFs (i.e., sterical hindrance; Courey and Jia, 2001; Daniels and Weis, 2005), initiating repressor complex formation (Courey and Jia, 2001; Ju et al., 2004), and influencing histone architecture/chromatin structure (Palaparti et al., 1997; Chen et al., 1999; Choi et al., 1999; Courey and Jia, 2001; Yochum and Ayer, 2001; Sekiya and Zaret, 2007). For a more in-depth review of long TLE/GRG structure and function, see Gasperowicz and Otto (2005) and Chen and Courey (2000).

The second TLE/GRG subgroup is composed of the Amino-terminal Enhancer of Split (AES) in humans and its mouse homolog GRG5 (Mallo et al., 1993; Miyasaka et al., 1993; Chen and Courey, 2000; Gasperowicz and Otto, 2005; Bajoghli, 2007). The AES/GRG5 proteins are truncated family members (relative to the long TLE/GRG members) as they consist only of the Q and GP domains (Fig. 1; Pinto and Lobe, 1996; Chen and Courey, 2000; Brantjes et al., 2001; Gasperowicz and Otto, 2005; Bajoghli, 2007). *AES/GRG5* is not an alternatively spliced variant of the long *TLE/GRG* gene but is a distinct family member expressed from its own locus (Mallo et al., 1993; Miyasaka et al., 1993; Mallo et al., 1995a; Gasperowicz and Otto, 2005; Bajoghli, 2007). There also exist truncated TLE/GRG family members that result from alternative splicing of the long *TLE/GRG* gene (Leon and Lobe, 1997; Lepourcelet and Shivdasani, 2002; Milili et al., 2002; Gasperowicz and Otto, 2005; Bajoghli, 2007) but their function is less clear and will not be discussed in this review. The Q domain of AES/GRG5 and the long TLE/GRG members both mediate multimerization between AES/GRG5 and/or long TLE/GRG proteins (Pinto and Lobe, 1996; Grbavec et al., 1998; Ren et al., 1999; Chen and Courey, 2000; Tetsuka et al., 2000; Brantjes et al., 2001; McLarren et al., 2001; Muhr et al., 2001; Daniels and Weis, 2005; Gasperowicz and Otto, 2005; Rave-Harel et al., 2005; Zhang et al., 2008, 2010) as well as interactions with TFs such as Tcf/Lef-1 (see Table 1; Brantjes et al., 2001; Daniels and Weis, 2005; Orian et al., 2007; Sekiya and Zaret, 2007). However, the GP domain of AES/GRG5 and long TLE/GRG members are conserved but functionally distinct, as AES/GRG5 does not interact with transcriptionally repressive HDAC proteins (HDAC-1 and -3; Brantjes et al., 2001; Yu et al., 2001; Gasperowicz and Otto, 2005; Bajoghli, 2007; Zhang et al., 2008). Because AES/GRG5 multimerizes with long TLE/GRG members but does not interact with HDACs, it is proposed that long TLE/GRGs lose their ability to form a functional, promoter/chromatin based tetrameric repressor complex (Pinto and Lobe, 1996; Palaparti et al., 1997; Chen et al., 1998; Choi et al., 1999; Chen and Courey, 2000; Brantjes et al., 2001; Yochum and Ayer, 2001; Gasperowicz and Otto, 2005). It is also possible that by oligomerizing with long TLE/GRGs (homotetramerization is required for long TLE/GRGs to interact with certain TFs) long TLE/GRGs lose their ability to interact with and repress certain TFs (Chen et al., 1998; Brantjes et al., 2001; Song et al., 2004; Daniels and Weis, 2005; Orian et al., 2007; Sekiya and Zaret, 2007). A potential result is that long TLE/GRGs are not recruited to the promoter through interaction with DNA-binding TFs (Fisher and Caudy, 1998; Cinnamon and Paroush, 2008). To date, no chromatin/DNA binding assays have been conducted to determine if AES/GRG5 localizes to the promoter (long TLE/GRG that possesses only the Q domain have been assayed; Sekiya and Zaret, 2007) or decreases long TLE/GRG:chromatin interaction. However, sequestration from the promoter/DNA-binding TF rather than antagonism of long TLE/GRGs at the promoter/chromatin is partially supported by transcriptional assays which show AES/GRG5 positively or negatively modulates general transcription if it is un-tethered or tethered to the DNA (Mallo et al., 1995b; Ren et al., 1999; Yu et al., 2001; Zhu et al., 2002). Although the mechanism(s) require further investigation/validation, in vitro, in situ, and/or in vivo data overexpressing/misexpressing AES/GRG5 show AES/GRG5 can antagonize transcriptional repression and/or physiological effects mediated by long TLE/GRGs in a context dependent manner (Roose et al., 1998; Chen and Courey, 2000; Wang et al., 2000; Brantjes et al., 2001; Muhr et al., 2001; Lopez-Rios et al., 2003; Swingler et al., 2004; Wang et al., 2004; Bajoghli et al., 2005; Gasperowicz and Otto, 2005; Rave-Harel et al., 2005; Allen et al., 2006; Bajoghli, 2007; Bajoghli et al., 2007; Zhang et al., 2008). Therefore, AES/GRG5 has been classified as a dominant-negative TLE/GRG family member (Chen and Courey, 2000; Brantjes et al., 2001; Muhr et al., 2001; Lopez-Rios et al., 2003; Swingler et al., 2004; Bajoghli et al., 2005; Gasperowicz and Otto, 2005; Rave-Harel et al., 2005; Allen et al., 2006; Bajoghli, 2007; Bajoghli et al., 2007; Zhang et al., 2008). However, cumulative analysis of the available studies (as will be discussed) suggests AES/GRG5 is a dynamic protein whose biological function is not exclusive to its defined role as a long TLE/GRG antagonist.

**Table 1:** Functional Interaction Between AES/GRG5 and TF/Non-TF Proteins^a^

AES/GRG5	TF/non-TF	Interaction domain of TF/non-TF	Interaction domain of AES/GRG5	Functional effect	Assay	Developmental/physiological effect	Refs
AES	Androgen receptor (AR)	N-terminus (aa. 1-559)	Q domain (aa. 1-129)	↓AR activity	Y2H, PD,CoIP RA, CFA	n/a	(Yu et al., 2001; Zhang et al., 2010)
AES	Bit1(non-TF)	n/a	n/a	↑Cytosolic Bit1-mediated apoptosis	Y2H, CoIP, CFA	Apoptosis	(Jan et al., 2004)
GRG5	mSix3, dSo (PD only), mSix6 (PD only)	eh-1 like motif in Six3 domain (aa. 1-183) conserved Phe-88 important	Q domain (1-134)	↑ mSix3 transcriptional repression	Y2H, PD, RA, ICC, no interaction via CoIP, AFA	Eye development	(Zhu et al., 2002)
GRG5	mSix2	No interaction	No interaction	n/a	PD	n/a	(Zhu et al., 2002)
AES*	Six3, Six6	Six domain for Six3 (∼ 1-205) and Six6 (1-127)	Q domain	↓ TLE-mediated Six3/6 transcriptional repression	Y2H, PD, ISH (me *-*AES and - Six3/6), AFA	Eye development	(Lopez-Rios et al., 2003)
AES	mSix2, *d*Optix	n/a	n/a	n/a	Y2H	n/a	(Lopez-Rios et al., 2003)
AES	Six1	n/a	n/a	n/a	Y2H, PD	n/a	(Lopez-Rios et al., 2003)
AES	mSix4	No interaction	No interaction	n/a	Y2H	n/a	(Lopez-Rios et al., 2003)
AES	PRDI-BF1	aa. 331-398	Q domain	Full length AES does not affect but truncated AES (ΔGP) inhibits PRDI-BF1- mediated activity	Y2H, PD, RA	n/a	(Ren et al., 1999)
AES	p65 subunit of NF-κB	Vicinity of p65 transactivation domain (aa. 477-521)	n/a	↓NF-κB-mediated activity	Y2H, PD, CoIP, RA	n/a	(Tetsuka et al., 2000)
GRG5*	mOct1	POU domain	Q domain	Potentially ↑ mOct1 activity	Y2H, PD, RA,	Potential↑ transcription of Gonadotropin releasing hormone	(Rave-Harel et al., 2005)
GRG5*	mMsx1	n/a	Q domain	↓ GRG1-mediated mMsx1 transcriptional repression	Y2H, PD, RA,	Potential↑ transcription of Gonadotropin releasing hormone	(Rave-Harel et al., 2005)
GRG5*, *x*AES*	Tcf1, *d*TCF, *x*TCF3	n/a	Q domain (aa. 1-106)	*x*AES and GRG5 ↑*x*Tcf3 activity	Y2H, PD, ICC, RA, and/or AFA	*x*AES ↑*x*Tcf3- mediated *Xenopus* axis duplication	(Roose et al., 1998; Brantjes et al., 2001)
GRG5, *x*AES	Lef1, mTcf3, mTcf4	No interaction	No interaction	n/a	Y2H	n/a	(Roose et al., 1998)
AES#	Lef1	n/a	n/a	↓ Lef1 activity	RA	n/a	(Arce et al., 2009)
AES*#	Lef1	n/a	n/a	↑ Lef1 activity	RA	n/a	(Beagle and Johnson, 2010)
GRG5*	mRunx2	Last 141 aa: VWRPY motif dispensable	Q and GP domain	↑ Runx2 activity	Y2H, CoIP, RA, AFA	Skeletal and growth development	(Wang et al., 2004)
AES*	PRH	No interaction	No interaction	↓ TLE1-mediated PRH transcriptional repression	CoIP, RA	n/a	(Swingler et al., 2004)
GRG5*	mNkx2.2, mNkx6.1	n/a	n/a	↓ GRG4-mediated mNkx -2.2 and -6.1 transcriptional repression	RA, AFA, ISH	Endogenous GRG5 not expressed during mNkx- mediated neuronal patterning	(Muhr et al., 2001)
GRG5	Hes1	No interaction	No interaction	Does not affect TLE- mediated Hes1 transcriptional repression	PD, CoIP, RA	n/a	(McLarren et al., 2001)
AES	LRP6-ICD (Non-TF)	n/a	n/a	LRP6-ICD ↓ AES-mediated LEF-1 activity	Y2H, CoIP, RA	n/a	(Beagle and Johnson, 2010)
AES*	HDRP (Non-TF)	aa. 178-343	First 17 aa dispensable	HDRP ↓ AES-mediated apoptosis	Y2H, PD, CoIP, CFA, RA	Apoptosis	(Zhang et al., 2008)
GRG5#	Pax5	n/a	n/a	Does not affect TLE- mediated Pax5 transcriptional repression		n/a	(Eberhard et al., 2000)
AES*#	rHNF3β	n/a	n/a	↑ HNF3β activity	RA	n/a	(Wang et al., 2000)
AES	TFIIE	n/a	n/a	Does not affect basal transcription of recombinant template	PD	n/a	(Yu et al., 2001)
AES	HDAC -1,-3	No interaction	No interaction		Y2H (HDAC1) PD (HDAC -1,-3)		(Yu et al., 2001; Zhang et al., 2008)
GRG5	HDAC1	No interaction	No interaction		CoIP		(Brantjes et al., 2001)

a Y2H, yeast-two-hybrid; RA, transcriptional reporter assay; PD, pull down; CoIP, co-immunoprecipitation assay; ISH, in situ hybridization; ICC, immunocytochemistry; AFA, functional assay in animal; CFA, cell culture based functional assay; TF/non-TF, Transcription Factor or non-Transcription Factor protein. Unless noted, the identified proteins are TFs. All TF/non-TF proteins are human unless noted and/or not identified by the study. d, *Drosophila*, me, medaka, x, *Xenopus*, r, rat, m, mouse.

* represent opposing effects between long TLE/GRGs and AES/GRG5 for the identified TF/non-TF protein.

# represent possible functional interactions for which interaction assays were not performed. n/a, information not reported or not identified/assayed.

Although significant data have accumulated in the past few years, AES/GRG5 remains a somewhat enigmatic protein. This is due in part to the varying models/systems, experimental procedures, signaling and developmental pathways and protein interactions used/analyzed across AES/GRG5 studies. Another confounding variable is the fact that almost all the studies use AES/GRG5 overexpression/misexpression. The overall focus of this review will be on various aspects of the AES/GRG5 protein including species conservation and evolution, expression and subcellular localization, protein interactions and its physiological role in development and disease. Although studies have been carried out on AES/GRG5 from various species, most have focused on human AES and/or mouse GRG5. Therefore, by default, this review will focus on human AES and/or mouse GRG5 unless noted.

## EVOLUTION AND CONSERVATION

**Fig. 2:**
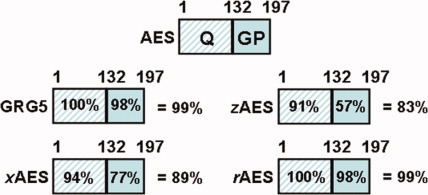
Conservation of AES protein sequence across species. Indicates percent homology of the Q domain, GP domain or entire AES protein for the indicated species relative to human AES. GRG5 is equivalent to mouse AES. *Xenopus (x)*, Zebrafish (z), Rat (r). Percent homology was calculated using global alignment with BLOSUM 62 scoring matrix. Amino acid sequence for the identified proteins are based upon the following NCBI accession numbers: AES (NP_001121.2), GRG5 (NP_034477.1), *r*AES (NP_062093.1), *x*AES (NP_001083532.1), *z*AES (NP_956717.1).

*AES/GRG-5* is highly conserved among mammals as well as other chordata such as *Xenopus* and zebrafish (Fig. 2; Chen and Courey, 2000; Bajoghli, 2007). The conserved nature of this protein is exemplified by the near perfect amino acid identity between human AES and other mammals such as mouse GRG5 (99% sequence identity) and the nonmammalian vertebrate *Xenopus* AES (89% sequence identity). This high degree of conservation increases the validity of extrapolating findings, especially functional interaction data, obtained with AES from one species (mostly human AES and/or mouse GRG5) in a more generalized context. However, studies which focus on potential AES/GRG5 interacting proteins sometimes use different isoforms and proteins from different species (see Table 1) that are not as highly conserved and thus caution needs to be exercised when generalizing about the functional outcomes. Finally, although “AES-like” proteins have been identified using the *Drosophilia* expressed sequence tag (EST) database (Chen and Courey, 2000), their existence and/or function has not been confirmed/investigated, to our knowledge.

Based upon a phylogenetic analysis of the different TLE/GRG subgroups, it is proposed *AES/GRG5* arose from tandem duplication of the long *TLE2/GRG2* gene (Bajoghli, 2007). Because AES/GRG5 is expressed throughout the phylum chordata (ex., human, mouse, zebrafish, *Xenopus*, etc), one can speculate that the duplication event took place before speciation. According to the classic model of gene duplication, the duplicate copy (i.e., *AES/GRG5*) persisted by acquiring key alterations/mutations in its GP domain coding sequence which allowed the AES/GRG5 protein to take on a new function (called neo-functionalization) as a dominant-negative member of the TLE/GRG family (described earlier; Bajoghli, 2007). Thus, it is speculated that AES/GRG5 evolved from the long TLE/GRG members (i.e., TLE2/GRG2) through mutation and neo-functionalization (Bajoghli, 2007).

## EXPRESSION AND LOCALIZATION

Analysis of endogenous AES/GRG5 shows the 25 kDa protein is broadly and constitutively expressed during development and adulthood with expression highest in muscle, heart, placenta (AES) and brain (GRG5; Mallo et al., 1993, 1995a; Miyasaka et al., 1993; Brantjes et al., 2001; Muhr et al., 2001; Zhu et al., 2002; Jan et al., 2004; Wang et al., 2004; Rave-Harel et al., 2005). However, developmental expression may be regulated temporally in a tissue and/or in a cell type dependent manner (Mallo et al., 1993, 1995a; Muhr et al., 2001). There are also data suggesting total AES/GRG5 expression is tightly regulated as GRG5 mice overexpressing high, but not moderate levels of GRG5, exhibit embryonic lethality (Allen et al., 2006). According to the NCBI Entrez database, three different transcripts/protein-isoforms have been identified for human AES: 1/a (1754 bp/266 amino acids; NP_945320.1), 2/b (1687 bp/197 amino acids; NP_001121.2) and 3/c (1684 bp/196 amino acids; NP_945321.1). Transcripts 2 and 3 were initially identified and differ slightly in their 5′ untranslated region (UTR) as well as an internal deletion of three nucleotides in transcript 3 (relative to transcript 2; Mallo et al., 1993; Miyasaka et al., 1993). *AES* is expressed from a single locus, therefore, it is speculated that the different transcripts result from alternative splicing and/or genetic polymorphism (Miyasaka et al., 1993). As a result of the internal tri-nucleotide deletion, protein isoform c (i.e., transcript 3) has a single glutamine deletion at residue 126 (residue number based upon isoform b sequence). Transcript/protein isoform 1/a is significantly longer and has a distinct N-terminus compared with isoforms b and c. Although 1/a is recognized as an AES variant in the NCBI Entrez database, there are no published data, to our knowledge, confirming its expression. In addition to the 197 amino acid mouse GRG5, a 202 amino acid isoform was also identified (Mallo et al., 1993; Miyasaka et al., 1993), but the 197 amino acid GRG5 is the only isoform registered in the NCBI Entrez database. Regardless, the 197 amino acid AES/GRG5 isoform is associated as the “canonical” AES/GRG5 protein (Choudhury et al., 1997; Ren et al., 1999; Chen and Courey, 2000; Tetsuka et al., 2000; Brantjes et al., 2001; Yu et al., 2001; Lopez-Rios et al., 2003; Gasperowicz and Otto, 2005; Zhang et al., 2008) and is the assumed isoform used by the studies referred to in this review.

In contrast to long TLE/GRG proteins, AES/GRG5 does not contain a putative nuclear localization signal (NLS) but is commonly referred to in the literature as a nuclear protein (Miyasaka et al., 1993; Mallo et al., 1995a; Chen and Courey, 2000; Gasperowicz and Otto, 2005). Such classification is likely do to initial studies showing endogenous GRG5 is exclusively nuclear in NB41A3 neuroblastoma cells (Mallo et al., 1995a), combined with the notion that AES/GRG5 functions as a transcriptional modulatory protein (i.e., nucleus). However, it has also been shown that exogenous AES can function in the cytosol (Jan et al., 2004) which suggests AES/GRG5 is not strictly a nuclear protein. Indeed, analysis of the studies on exogenous and/or endogenous AES/GRG5 (as well as *Xenopus* AES) suggests subcellular distribution is influenced by cell type as it is exclusively nuclear in some cells including gonadotropin hormone releasing neurons (Mallo et al., 1995a; Zhu et al., 2002; Rave-Harel et al., 2005; Zhang et al., 2008), cytosolic in COS-7 cells (Cavallo et al., 1998; Roose et al., 1998) and nucleocytoplasmic in cells including HEK 293T cells (Jan et al., 2004; Beagle and Johnson, 2010). AES/GRG5 lacks a putative nuclear export signal (NES) or NLS and, therefore, regulation by the importin or exportin family of proteins seems unlikely (Ullman et al., 1997). Although its relatively small size (∼25 kDa) allows for nuclear diffusion (Liu et al., 2005), it is reasonable to assume the transcriptional activity of various TFs is not regulated by the random diffusion of effector proteins such as AES/GRG5. Therefore, what are the underlying mechanism(s) that regulate AES/GRG5 subcellular distribution? Using *Xenopus* AES/GRG5 in COS-7 cells (89% sequence identity to human AES, see Fig. 2), one study showed subcellular distribution is regulated by certain Tcf (but not Lef-1) TFs (Roose et al., 1998). It is speculated that AES/GRG5 subcellular distribution might also be mediated by fellow long TLE/GRG family members (Chen and Courey, 2000). This suggests AES/GRG5 localization is context-dependent, therefore, differential expression of AES/GRG5 interacting proteins (ex. Tcf or long TLE/GRG; Mallo et al., 1995a; Chen and Courey, 2000; Brantjes et al., 2001) might explain why its subcellular distribution varies across cell lines. As will be discussed, AES/GRG5 can modulate the activity of various TF/non-TF proteins (see Table 1). Therefore, AES/GRG5 localization may serve as a regulatory mechanism by mediating AES/GRG5's ability to colocalize with and modulate various TF/non-TF proteins. Indeed, our lab has shown that localization in part regulates exogenous AES's ability to positively modulate Lef-1 transcriptional activity in HEK 293T cells (Beagle and Johnson, 2010). While further studies are required to elucidate the regulatory mechanism(s) that mediate endogenous AES/GRG5 subcellular distribution, the data strongly suggest that AES/GRG5 should not be classified, in general, as a nuclear protein.

## PROTEIN INTERACTIONS

Table 1 (adapted from Gasperowicz and Otto, 2005) outlines the various functional interactions, or lack thereof, that have been identified between TF/non-TF proteins and AES/GRG5 (as well as other AES species). Table 1 also includes a brief description, when possible, summarizing how the identified interactions effect the TF/non-TF protein activity and/or AES/GRG5 activity as well as the physiological/biological affect. It should be noted that all functional interaction data are based upon overexpression/misexpression of AES/GRG5 and, therefore, caution must be used in extrapolating to physiological conditions. Although both long TLE/GRG and AES/GRG5 protein(s) interact with many of the same proteins, how they interact with, as well as the ability to interact with certain TF/non-TF proteins differs. As expected, TFs that interact exclusively with the C-terminal SP and WD40 domains of the long TLE/GRGs, do not interact with AES/GRG5 due to the lack of these domains (Chen and Courey, 2000; Eberhard et al., 2000; McLarren et al., 2001; Gasperowicz and Otto, 2005; Bajoghli, 2007). However, there are TFs such as the androgen receptor (AR) and Six4 that can interact with the Q domain of AES but not the Q domain of a full-length long TLE-2 or -3 protein (Lopez-Rios et al., 2003; Zhang et al., 2010). Subsequently, it was shown the C-terminal domains (CcN, SP, and/or WD40) of long TLE/GRG proteins actually block interaction with such TFs as AR and Six4 (Lopez-Rios et al., 2003; Zhang et al., 2010). Similarly, a series of elegant experiments involving AES-mediated AR repression also revealed a novel intramolecular regulatory mechanism for AES (Zhang et al., 2010). First, it was shown that intermolecular AES homodimerization, mediated by residues 1–129 of the Q domain, is required for it to interact with and inhibit AR transcriptional activity. This homodimerization domain located within the AES protein (classified as the AR inhibitory domain) can intramolecularly interact w/ residues 156–176 of AES (classified as the negative regulatory domain) and prevent intermolecular homodimerization between AES proteins. However, residues 190–193 of AES (classified as the positive regulatory domain) prevents intramolecular interaction between residues 1–129/156–176 within the AES protein (i.e., AR inhibitory domain/negative regulatory domain) thereby allowing AES intermolecular homodimerization and AR repression. This is an important finding as Q domain-mediated homo- and/or hetero-oligomerization is believed to play a critical role in AES/GRG5s ability to antagonize long TLE/GRGs as well as positively modulate transcription factors such as Tcf/Lef-1 (see Introduction for more details; Roose et al., 1998; Brantjes et al., 2001; Daniels and Weis, 2005; Beagle and Johnson, 2010). Future studies should be conducted to determine if the described intramolecular regulatory mechanism is a general or TF/non-TF specific AES regulatory mechanism.

Interestingly, data suggest that AES/GRG5 does not act as a general negative regulator of long TLE/GRGs as neither GRG5 nor AES antagonize long GRG4-mediated Pax5 (Eberhard et al., 2000) or long TLE-mediated Hes1 (McLarren et al., 2001) transcriptional repression. Because AES/GRG5 does not interact with TFs such as Hes1 (McLarren et al., 2001) it is speculated AES/GRG5 might only exert a dominant-negative effect over long TLE/GRGs when both family subgroups can interact with specific DNA-binding TFs, such as Runx2 (Thirunavukkarasu et al., 1998; Wang et al., 2004). However, this notion conflicts with data showing both AES and long TLE1 can interact with and repress NF-κB-mediated transcriptional activity (Tetsuka et al., 2000). Also, GRG5 can antagonize long GRG-mediated PRH transcriptional repression even though GRG5 does not interact with PRH (Swingler et al., 2004). These examples clearly show that the mechanism(s) by which AES/GRG5 antagonizes long TLE/GRGs activity needs to be investigated further.

Such examples also serve to illustrate the fact that AES/GRG5, unlike the long TLE/GRG proteins, is not a dedicated TF co-repressor. In contrast, AES/GRG5 can positively or negatively modulate various TFs as well as non-TF proteins (see Table 1). The fact that AES can repress TFs such as AR (Yu et al., 2001; Zhang et al., 2010) and NF-κB (Tetsuka et al., 2000) clearly shows that AES/GRG5 does not function exclusively as an antagonist of long TLE/GRG repressor activity. For example, AES but not full length long TLE-2 or -3, mediates AR repression by preventing AR:DNA interaction (Zhang et al., 2010). AES/GRG5 might also be capable of directly modulating transcription independent of long TLE/GRG family members as AES was shown to interact with TFIIE, a basal TF (Yu et al., 2001). The functional impact of an AES:TFIIE interaction has not been explored but it was speculated that AES negatively regulates basal transcription through TFIIE interaction (Yu et al., 2001). However, the same study also showed AES enhanced GAL4-VP16-mediated transcription of a recombinant template that required TFIIE (in addition to other basic transcriptional components). Further, AES/GRG5 also plays a role in cellular processes not directly associated with transcription, unlike the long TLE/GRG proteins. By interacting with cytosolic Bit1, a proapoptotic protein, AES was shown to promote anoikis (i.e., cell detachment)-mediated apoptosis (Jan et al., 2004). Taken as a whole, data suggest AES/GRG5 is a multifunctional protein whose biological activity is not based solely upon its ability to antagonize long TLE/GRG proteins.

In addition to localization, AES/GRG5 activity is regulated through interactions with non-TF proteins such HDAC-related protein (HDRP; Zhang et al., 2008) and the soluble intracellular domain of the LRP6 receptor (LRP6-ICD; Beagle and Johnson, 2010). HDRP (an HDAC1 interacting protein) does not affect AES localization but may repress AES dominant-negative activity over long TLE1 thereby allowing long TLE1-mediated repression of proapoptotic genes (Zhang et al., 2008). Because AES does not interact with HDAC1 (Yu et al., 2001; Zhang et al., 2008), it also suggest that AES does not exist in a complex with HDRP:HDAC1. Therefore, it would be interesting to see how AES effects the transcriptional repressive activity mediated by the HDRP:HDAC1 interaction. Nonetheless, data suggest that AES/GRG5 activity is also regulated, in addition to localization, through interaction with other proteins (Zhang et al., 2008; Beagle and Johnson, 2010).

While informative, most functional interaction data are derived from cell culture and animal based assays involving overexpression/misexpression of exogenous AES/GRG5. However, AES/GRG5 developmental expression is temporally restricted in a cell type/tissue dependent manner (Mallo et al., 1993, 1995a; Muhr et al., 2001), therefore, the physiological effect mediated by certain functional interactions may not be relevant. For example, GRG4-mediated Nkx transcriptional repression establishes progenitor cell pattern and neuronal fate in the ventral neural tube, a phenotype defined in a temporally relevant manner (Muhr et al., 2001). Overexpressing/misexpressing GRG5 can mitigate the neuronal phenotype by antagonizing GRG4-mediated Nkx transcriptional repression but the same study also showed endogenous GRG5 is not developmentally expressed at the relevant time point (Muhr et al., 2001). Therefore, the potential physiological effect of AES/GRG5-mediated functional interactions should be carefully examined in the context of endogenous protein expression in a physiologically relevant context.

## ROLE IN DEVELOPMENT AND DISEASE

The TLE/GRG family mediates expression of numerous developmental genes that are regulated by transcriptional signaling pathways such as the Wnt/β-catenin pathway (Chen and Courey, 2000; Brantjes et al., 2001; Bajoghli et al., 2005, 2007; Gasperowicz and Otto, 2005; Buscarlet and Stifani, 2007; Cinnamon and Paroush, 2008). As a result, the TLE/GRG family plays an essential role in numerous developmental processes. AES/GRG5 activity has been implicated in developmental processes including hematopoeisis (Swingler et al., 2004), ear development (Aghaallaei et al., 2005; Bajoghli et al., 2005), heart formation (Bajoghli et al., 2007), puberty (Rave-Harel et al., 2005), pituitary gland development (Brinkmeier et al., 2003), and *Xenopus* axis formation (Roose et al., 1998) to name a few. Although the above processes have been tentatively studied, the majority of in vivo studies have focused on the functional role of AES/GRG5 in growth and osteogenesis, as well as eye development, which we will discuss below.

The Runx2 TF is required for differentiation and function of osteoblast (Wang et al., 2002, 2004). Interaction and functional assays in cell culture and *Runx2^+/−^GRG5^−/−^* mice suggest GRG5 enhances Runx2 transcriptional activity in vivo to regulate postnatal growth in mice (Wang et al., 2004). The lack of GRG5 activity significantly potentiated defective membranous bone formation in *Runx2* heterozygotes and caused a severe long bone growth plate defect. The bone and cartilage defects were associated with reduced Indian hedgehog (Ihh) expression which mediates bone and cartilage development (Wang et al., 2002, 2004). The developmental defects and reduced Ihh activity were only seen in *Runx2^+/−^GRG5^−/−^* (not *Runx2^+/−^GRG5^+/+^*) mice which suggest GRG5 effects Ihh activity through Runx2 (Wang et al., 2002, 2004). In addition to Runx2, AES/GRG5 might also influence postnatal skeletal growth through Tcf4 and Lef1 which are expressed in skeletal tissue and whose activity is modulated by AES/GRG5 and the long TLE/GRGs (Roose et al., 1998; Hartmann and Tabin, 2000; Brantjes et al., 2001; Daniels and Weis, 2005; Cinnamon and Paroush, 2008; Beagle and Johnson, 2010). Regulation of AR activity may serve an additional mechanism by which AES/GRG5 can affect postnatal growth properties (skeletal and/or nonskeletal) (Yu et al., 2001; Frenkel et al., 2010; Zhang et al., 2010).

Six3 and Six6 are DNA-binding TFs required for eye development, mediated in part by interaction with TLE/GRG proteins (Kobayashi et al., 2001; Zhu et al., 2002; Lopez-Rios et al., 2003). AES and GRG5 (in addition to Groucho, long TLE1 and/or GRG4) were shown to interact with Six3 and/or Six6 by means of GST pulldown and yeast two hybrid studies (Zhu et al., 2002; Lopez-Rios et al., 2003). Although *Drosophilia* Groucho and long GRG4 were shown to interact with Six3 by means of co-immunoprecipitation in NIH3T3 mammalian cells, this interaction could not be detected for GRG5 (Zhu et al., 2002). Expression patterns of exogenous and/or endogenous medaka (rice fish) AES or GRG5 was also shown to overlap with Six3 and Six6 in the developing medaka eye (Lopez-Rios et al., 2003) or Six3 in the mouse embryo as well as colocalize in NIH3T3 cells (Zhu et al., 2002). Through modulation of Six3 and/or Six6, in vivo data suggest exogenous AES/GRG5 activity plays a critical role in certain aspects of eye development (Zhu et al., 2002; Lopez-Rios et al., 2003). Reporter assays show that GRG5 can enhance Six3 activity which is required for early eye development (Zhu et al., 2002). Although no difference in lens morphogenesis and crystallin regulation was seen between chick embryos overexpressing Six3 alone or with GRG5, there are data suggesting GRG5 can modulate Six3-mediated retinogenic formation in postnatal mice (Zhu et al., 2002). It should be noted that in this study, a mutant Six3 expression construct incapable of binding either long GRGs or GRG5 was used and thus these findings require further validation (Zhu et al., 2002). The observation is supported however in medaka fish overexpressing human AES which showed AES can modulate Six3- and Six6-mediated expression of ectopic retina tissue (Lopez-Rios et al., 2003). The cell culture and in vivo expression patterns, interaction and functional assays strongly suggest AES/GRG5 modulates eye development by means of Six3 and/or Six6. However, additional functional assays involving endogenous AES/GRG5 are required to better define AES/GRG5s role in eye development and its role relative to that of the long TLE/GRGs.

Similar to long TLE/GRGs, AES/GRG5 may be involved in nondevelopmental processes, as it has been implicated in various pathological conditions including cancer. For example, a *GRG1/GRG5* transgenic mouse model showed GRG5 overexpression reduced tumor burden due to GRG1 overexpression induced lung adenocarcinoma (Allen et al., 2006). Furthermore, AES functions as a proapoptotic protein (in a context dependent manner) by antagonizing the antiapoptotic effects mediated by TLE1 (Jan et al., 2004; Zhang et al., 2008), whose elevated expression is associated with certain cancers such as lymphoma (Shipp et al., 2002). In addition to its role in osteogenesis, Runx2, a TF positively or negatively modulated by GRG5 or long TLE/GRGs (Thirunavukkarasu et al., 1998; Wang et al., 2004; Gasperowicz and Otto, 2005), functions as a potent tumor suppressor (Baniwal et al., 2009). Negative modulation of AR activity, whose aberrant activity is commonly associated with prostate cancer, similarly suggests a role for AES/GRG5 in certain cancers independent of its dominant-negative activity over long TLE/GRG proteins (Yu et al., 2001; Baniwal et al., 2009; Zhang et al., 2010). Taken as a whole, the data reveal a potential anti-oncogenic function for AES/GRG5 in a long TLE/GRG-dependent (i.e., dominant-negative activity) as well as -independent manner. The ability to influence osteocyte activity through Runx2 (and potentially AR) indicates AES/GRG5 can influence other pathological processes including bone disorders such as osteoporosis (Baniwal et al., 2009; Frenkel et al., 2010). Regulation of immunomodulatory TFs such as NF-κB (Tetsuka et al., 2000) and PRDI-BF1 (Ren et al., 1999) suggest an immunological function for AES/GRG5, a potential physiological role that can be further explored in the various transgenic mouse models used in the field of immunology.

The described functional data strongly suggest AES/GRG5 imparts significant and broad physiological activity in both development and adulthood. However, extreme caution is required when interpreting the animal and functional interaction data. First, the GRG5 mice, generated by targeted disruption of the GRG5 allele, exhibit only transient growth retardation (Mallo et al., 1995a; Wang et al., 2002, 2004). While one study showed 20% of GRG5 null mice exhibit growth retardation severe enough to cause death (Mallo et al., 1995a), data show most GRG5 null mice are viable and their overall growth is at least 80% that of their control littermates (Mallo et al., 1995a; Wang et al., 2002, 2004). In the absence of decreased co-factor expression (ex. Runx2), the data suggest GRG5 is dispensable for appropriate terminal growth (i.e., adult body weight) as well as terminal skeletal and chondral development. It is interesting to note that the effect on terminal growth as well as growth rate relative to the control littermates was less severe for the female GRG5 null mice compared with the GRG5 null male mice (Wang et al., 2002, 2004). Reasons for the gender difference remain to be investigated. While GRG5 null mice are fertile (Mallo et al., 1995a; Wang et al., 2002; although *Runx2^+/−^GRG5^−/−^* mice are infertile; Wang et al., 2004) with some abnormal reproductive behavior (Mallo et al., 1995a), most studies focused only on body weight, skeletal, and/or chondral properties. One of the studies did not find any reproducible pathology in the GRG5 null mice upon “extensive histopathological analyses” but a description of the analyses was not provided (Mallo et al., 1995a). However, one GRG5 null study focusing exclusively on mouse pituitary development showed abnormal pituitary gland enlargement (Brinkmeier et al., 2003). Unfortunately, the study only focused on embryonic day (E) 14.5, E16.5 and postnatal day 1, therefore, the long-term consequences, if any, on pituitary development and/or function are unknown. As a result, it is not clear what effect, if any, loss of GRG5 has on the phenotype of other traits (ex. immune function, tissue homeostasis, etc) in development and/or adulthood. Second, the described functional interactions and/or effects mediated by AES/GRG5 are based upon exogenous AES/GRG5 that is overexpressed/misexpressed in various cell culture and/or animal based assays. Therefore, to determine the physiological relevance of AES/GRG5 in development and/or adulthood, caution must be used when critically examining the current research. With that said, the ubiquitous expression of endogenous AES/GRG5 combined with the described functional data does suggest AES/GRG5 is a physiologically relevant protein. Targeted inactivation of genes such as retinoic acid receptor-α (Li et al., 1993; Cammas et al., 2010) and transglutaminase 2 (De Laurenzi and Melino, 2001; Nanda et al., 2001), thought to be important for a wide variety of functions, have very mild or even no phenotypic consequences. For many of the described AES/GRG5 functional interactions such as NF-κB (Tetsuka et al., 2000) and Runx2 (Baniwal et al., 2009), the interacting TF/non-TF activity is regulated by other factors. The lack of an obvious terminal phenotype in the GRG5 null mice might, therefore, be a result of functional redundancy mediated by other regulatory/interacting protein. Another plausible reason (not mutually exclusive) is AES/GRG5 may not be acting as a primary controller, but rather as a modulatory regulatory protein. AES/GRG5's physiological impact may, therefore, be more subtle and not as readily elucidated. Determining AES/GRG5's physiological significance will ultimately require analysis of endogenous AES/GRG5 in a physiologically relevant manner.

## PERSPECTIVE

The TLE/GRG family consists of transcriptional modulatory proteins that play a critical role in numerous developmental as well as homeostatic processes. While the transcriptional repressive function of long TLE/GRGs has been extensively defined (relative to AES/GRG5), the dynamic properties exhibited by AES/GRG5 make it a more difficult protein to classify. AES/GRG5 is classically identified as a dominant-negative TLE/GRG family member that functions to antagonize long TLE/GRG repressor activity. However, a review of the functional interactions modulated by AES/GRG5 overexpression/misexpression shows AES/GRG5 can also function in a long TLE/GRG independent manner. In contrast to the long TLE/GRG proteins which function as dedicated transcriptional repressors, the reviewed data show AES/GRG5 can influence transcriptional and nontranscriptional events by positively or negatively regulating/effecting/modulating various TF and non-TF proteins (see Table 1). Cumulative analysis of the available data, therefore, strongly suggests AES/GRG5's functional activity should not be relegated to its dominant-negative classification.

The context-dependent dominant-negative activity of AES/GRG5 has well been studied, yet the mechanism by which AES/GRG5 antagonizes long TLE/GRGs is not clear and requires further investigation/validation. Is AES/GRG5 sequestering long TLE/GRGs from the promoter based TF and/or inhibiting the promoter/chromatin based repressor function of long TLE/GRGs through lack of HDAC recruitment? What additional mechanism(s) does AES/GRG5 modulate the transcriptional activity of TFs in a manner independent of long TLE/GRG antagonism? Can AES/GRG5 influence transcription through the basal transcriptional machinery (i.e., TFIIE), effect recruitment of co-activator or -repressor complexes and/or effect TF promoter binding? Delineation of such questions should help clarify and more accurately classify AES/GRG5s functional role within the TLE/GRG family as well as its context dependent cellular function.

AES/GRG5 is unique in that it can functionally interact with cytosolic and nuclear localized TF/non-TF proteins. Although endogenous AES/GRG5 expression is ubiquitous, its subcellular distribution varies in a cell type dependent manner and that differential localization functions in part, to regulate AES/GRG5 activity. There are data suggesting AES/GRG5 localization is mediated by proteins whose activity in turn is modulated by AES/GRG5. Therefore, it is possible that differential expression of such proteins explains the cell type dependent subcellular distribution of AES/GRG5. Although the regulatory mechanism(s) that mediate its localization requires significant investigation and validation, the data clearly show AES/GRG5 subcellular classification should be defined according to cell type as opposed to its classic description as a nuclear protein.

In conclusion, AES/GRG5 is capable of functionally interacting with numerous TF/non-TF proteins and, therefore, has the potential to influence numerous cellular/physiological processes. There are data suggesting AES/GRG5 in conjunction with other co-factors plays a critical role in developmental processes such as growth and eye development as well as pathological processes including cancer. Although informative, the described studies almost exclusively involve cell culture and animal based assays in which exogenous AES/GRG5 is being overexpressed/misexpressed. While endogenous AES/GRG5 expression is widespread in the adult, future AES/GRG5 studies should confirm the interaction and/or co-expression between endogenous AES/GRG5 and interacting TF/non-TF proteins as well as the physiological process being studied (if applicable). This is especially important when analyzing the developmental effects mediated by AES/GRG5 as expression is temporally restricted.
